# Self-Assembling Nanovaccine Confers Complete Protection Against Zika Virus Without Causing Antibody-Dependent Enhancement

**DOI:** 10.3389/fimmu.2022.905431

**Published:** 2022-05-09

**Authors:** Heng Rong, Mi Qi, Jingdi Pan, Yuhan Sun, Jiawang Gao, Xiaowei Zhang, Wei Li, Bo Zhang, Xian-En Zhang, Zongqiang Cui

**Affiliations:** ^1^State Key Laboratory of Virology, Wuhan Institute of Virology, Center for Biosafety Mega-Science, Chinese Academy of Sciences, Wuhan, China; ^2^University of Chinese Academy of Sciences, Beijing, China; ^3^Faculty of Synthetic Biology, Shenzhen Institutes of Advanced Technology, Chinese Academy of Sciences, Shenzhen, China

**Keywords:** zika virus, nanovaccine, ferritin, ZIKV envelop protein domain III, antibody-dependent enhancement

## Abstract

The Zika virus (ZIKV) epidemic poses a substantial threat to the public, and the development of safe and effective vaccines is a demanding challenge. In this study, we constructed a kind of self-assembling nanovaccine which confers complete protection against ZIKV infection. The ZIKV envelop protein domain III (zEDIII) was presented on recombinant human heavy chain ferritin (rHF) to form the zEDIII-rHF nanoparticle. Immunization of mice with zEDIII-rHF nanoparticle in the absence of an adjuvant induced robust humoral and cellular immune responses. zEDIII-rHF vaccination conferred complete protection against lethal infection with ZIKV and eliminated pathological symptoms in the brain. Importantly, the zEDIII-rHF nanovaccine induced immune response did not cross-react with dengue virus-2, overcoming the antibody-dependent enhancement (ADE) problem that is a safety concern for ZIKV vaccine development. Our constructed zEDIII-rHF nanovaccine, with superior protective performance and avoidance of ADE, provides an effective and safe vaccine candidate against ZIKV.

## Introduction

Zika virus (ZIKV) is an arboviral virus belonging to the *Flaviviridae* family. ZIKV is defined as a serious public health problem by the World Health Organization (WHO) because it is widespread in many countries, with cases of severe birth defects being documented (1, 2). ZIKV also causes severe neurological diseases, such as microcephaly, Guillain–Barré syndrome, meningoencephalitis, and myelitis (3, 4). Although control and preventive measures have been taken, to date, there are no vaccines or specific antiviral drugs against ZIKV.

Several platforms have been tried to develop ZIKV vaccines. For example, live attenuated ZIKV vaccine candidates have been generated by deleting 10 nucleotides in the 3’-untranslated region (UTR) of the ZIKV genome or using the codon pair deoptimization strategy (5). An inactivated full-virus ZIKV vaccine was also developed and induced protection against ZIKV infection (6). However, attenuated live vaccines have hidden dangers, such as infectious residues, and inactivated ZIKV causes immune-related side effects (7). As the envelope (E) protein and NS1 protein are major targets of host antibody responses, they were also considered candidates for ZIKV vaccines. Li et al. developed an attenuated recombinant vesicular stomatitis virus (rVSV) expressing a ZIKV prM-E-NS1 polyprotein (8). This rVSV could induce ZIKV-specific antibodies and a T cell immune response and protect mice against ZIKV infection. DNA or RNA vaccination based on the ZIKV prM-E gene sequence could also induce strong neutralizing antibodies (NAbs) and a T cell immune response and effectively improve the survival rate in mice (9). However, for these candidates, due to the complex preparation processes and stringent storage conditions, there are obstacles limiting large-scale production (10). More importantly, these E protein-based vaccines may cause antibody-dependent enhancement (ADE) and have the potential risk of enhancing other flavivirus infections (11, 12).

The nonneutralizing cross-reactive antibodies generated during a previous flavivirus infection can increase the pathogenesis of a related virus, which is called ADE (13). ADE is particularly common between ZIKV and dengue virus (DENV) (14, 15). ADE is a challenge in vaccine development for flaviviruses, including ZIKV. Approaches to ensure high protective efficacy while avoiding ADE are an important focus in the development of ZIKV vaccines (15). Recently, it was found that ZIKV E protein domain III (zEDIII) can evoke ZIKV-specific antibody and NAb responses without ADE activity for DENV infection (16, 17). Thus, vaccines based on the zEDIII antigen are potential protein subunit vaccine candidates for ZIKV infection. However, the zEDIII subunit has low immunogenicity (18), which limits it to be developed as protective vaccine.

Self-assembling nanotechnology provides an opportunity for the development of vaccines with superior performance (19–21). Nanoparticles can promote antigen delivery and immune induction (22–25). By presenting the influenza A virus (IAV) trimeric HA or M2e on self-assembling ferritin, nanoparticle vaccines have been developed to confer influenza protection (26). Other nanoparticle vaccines have been tried to prevent Dengue virus and Hepatitis B virus (27, 28). Recently, nanoparticle-based vaccine against SARS-CoV-2 was also reported (29). These nanoparticle vaccines cause more efficacious immune response and protection, which provides a promising strategy for vaccine construction.

In this study, we developed a self-assembling nanovaccine to protect against ZIKV infection. By displaying ZIKV zEDIII on the recombinant human heavy chain ferritin (rHF) cage, we created zEDIII-rHF nanoparticles. Immunization of mice with zEDIII-rHF nanoparticles in the absence of an adjuvant induced robust immune responses, including humoral and cellular immune responses. Vaccination with zEDIII-rHF nanoparticles also conferred complete protection against lethal infection with ZIKV and avoided ADE of infection with a heterologous flavivirus (i.e., DENV). Our study provides an effective and safe nanovaccine against ZIKV.

## Materials and Methods

### Viruses and Cells

An Asian-lineage ZIKV strain (ZIKV SZ-WIV01 GenBank: KU963796.1) was provided by the China Centre for General Virus Culture Collection (CCGVCC) (30). ZIKV was propagated in Vero cells and quantified by the TCID_50_. Vero cells (CCL-81, ATCC) were cultured in Dulbecco’s modified Eagle’s medium (DMEM; Gibco, Carlsbad, CA, USA) containing 10% fetal bovine serum (FBS; Gibco, Carlsbad, CA, USA), 100 U/ml penicillin and 100 μg/ml streptomycin and maintained in 5% CO_2_ at 37°C. THP-1 cells (TIB-202, ATCC) were obtained from Professor Hanzhong Wang at the Wuhan Institute of Virology, Chinese Academy of Sciences. THP-1 cells were cultured in Roswell Park Memorial Institute medium (RPMI-1640; Gibco, Carlsbad, CA, USA) containing 10% FBS (Gibco, Carlsbad, CA, USA), 100 U/ml penicillin and 100 μg/ml streptomycin and maintained in 5% CO_2_ at 37°C (19).

### Generation of Vectors Expressing zEDIII-rHF

pET28a-zEDIII-rHF was constructed using the zEDIII (SZ-WIV01) sequence and recombinant human heavy chain ferritin sequence (rHF, Gene ID: 2495). zEDIII-rHF was expressed in *E. coli* and purified with a nickel column that recognizes the 6-His-tag. The protein was further purified *via* Size Exclusion Chromatography (GE Healthcare, Uppsala, Sweden) on a Superose 6 column. The purified protein was quantified by a BCA protein assay kit (Beyotime, China), and the endotoxin concentration was tested with the ToxinSensor Single Tests Kit (GenScript, USA).

### SDS-PAGE, Western Blotting, TEM, and DLS

SDS-PAGE and western blotting were used to characterize the size, identity, and purity of recombinant zEDIII-rHF. The antibody was an anti-zEDIII antibody (Sino Biological Cat: 40543-R101).

The shape and size of zEDIII-rHF and rHF particles were characterized by using transmission electron microscopy (TEM) (FEI Tecnai G220 TWIN electron) and dynamic light scattering (DLS) (Zetasizer Nano ZS ZEN3600, Malvern Instruments Ltd.). For the TEM, samples were absorbed onto carbon-coated copper grids for two minutes, and then were negatively stained with 2.0% phosphotungstic acid (PTA, PH 7.0) for ten minutes. The grids were dried in atmosphere and checked with transmission electron microscopy. Zeta potentials were measured by the Zetasizer Nano ZS ZEN3600.

### Animal Immunization

ZIKV-adapted mice (B6.129S2-Ifnar1^tm1Agt^) were a gift from Professor Genhong Cheng (The University of California, Los Angeles (UCLA)) (31). Animals were raised under specific pathogen-free (SPF) conditions and fed sterile food and water. Six-week-old B6.129S2-Ifnar1^tm1Agt^ mice were divided into 4 groups (n=5 per group). The mice in Group 1 received PBS as a mock-immunized control. Those in Group 2 and Group 3 received 3.75 µg of zEDIII protein (zEDIII molar equivalent) or 6.25 µg of rHF nanoparticles, respectively. The mice in Group 4 received 10 µg of zEDIII-rHF protein (zEDIII molar equivalent). The primary immunization date was set as day 0. The mice were boosted on days 14 and 28. Blood samples were collected from the retro-orbital vein on days 10, 24 and 38, and sera were isolated for antibody detection with an indirect enzyme-linked immunosorbent assay (ELISA). The highest serum dilution that gave an OD_450_ of more than twice that of the lowest serum dilution of the PBS group was regarded as the endpoint of the antibody titer.

### Flow Cytometry and ELISPOT Assays

Mice were euthanized, and the spleen was aseptically removed for *in vitro* splenocyte culture. Lymphocytes were isolated by using mouse lymphocyte separation medium (Dakewei, Beijing, China). Flow cytometry was used to measure the percentages of CD4^+^ and CD8^+^ T cells in splenic lymphocytes. We labeled cells with fluorophore-conjugated antibodies including anti-CD3 (APC/Cy7, Dakewei), anti-CD4 (FITC, Dakewei), and anti-CD8 (PerCP, Dakewei). An LSRFortessa flow cytometer (BD Biosciences) and FlowJo software (TreeStar) were used for data collection and analysis, respectively. ELISPOT was used to evaluate the numbers of IL-4- and IFN-γ-secreting cells. ELISPOT assay kits were used according to the manufacturer’s instructions (DKW22-2000-096/DKW22-2040-096). A total of 10^5^ lymphocytes were added to each well in triplicate and stimulated with 1 µg of zEDIII.

### Neutralization Assay

The PRNT_50_ was applied to measure zEDIII-specific NAbs (32). Blood samples were collected from the retro-orbital vein of mice in different groups. Serum samples were treated with 56°C water for 30 minutes. The serum samples were diluted along a gradient, mixed with 100 PFU of ZIKV in the same volume and incubated at 37°C for 1.5 h. The mixture was incubated with cells for another 1.5 h at 37°C, the supernatant was removed, and the cells were washed with PBS. A 1.25% methyl cellulose overlay was added, and the plates were incubated for 4 days in 5% CO_2_ at 37°C. Then, the cells were fixed with 4% paraformaldehyde and stained with 0.5% crystal violet. Plaque morphology and numbers were recorded after rinsing the plates with deionized water. Samples with titers of ≥10 were considered to be seropositive.

### ZIKV Challenge and qRT-PCR Assays

The LD_50_ was calculated using the Reed & Muench method. To evaluate the protective effect of zEDIII-rHF nanoparticles against ZIKV, 14 days after the final immunization, each mouse was infected with 20 µl of ZIKV (10× LD_50_ dose, SZ-WIV01) by foot pad injection. Over the next 21 days, the mice were monitored for body weight loss and survival. Body weight loss of more than 20% was considered indicative of lethality. Mice were euthanized according to the study guidelines. The viral challenge experiments were carried out in an ABSL-2 facility. Blood was collected from all mice for viremia analysis on day 3 and day 7 postinfection. The brain, testis, kidneys, spleen, and liver were collected for viral titer detection. Serum and tissue samples were obtained from mice, and total RNA was extracted with an RNA extraction reagent kit (Magen Cat: R4173-03). A universal pair of primers (forward primer: 5’-AGGATCATAGGTGATGAAGAAAAGT-3’ and reverse primer: 5’-CCTGACAACACTAAGATTGGTGC-3’) was used to amplify ZIKV (33). All qRT-PCR assays were performed with the One-step TB Green PrimeScript™ PLUS RT-PCR Kit (TaKaRa, Code No. RR096A) on a CFX96 Touch real-time PCR detection system (Bio-Rad).

### ADE Assays

DENV-2 challenge was carried out as previously described, and blood samples were collected for RT-PCR analysis on day 3 and day 7 post infection. A universal pair of primers (forward primer: 5’-GAAGACATTGACTGYTGGTGCAA-3’ and reverse primer: 5’-CGATGTTTCCACGCCCCTTC-3’) was used to amplify DENV-2 (34). THP-1 cells were treated with 5 ng/ml phorbol 12-myristate 13-acetate (PMA) and induced to differentiate into macrophages for the ADE experiment. Immunized mouse sera were obtained, treated by heat inactivation, mixed with DENV-2 and incubated for 1 h at 37°C. Then, the mixture was added to THP-1 cells that expressed an Fc-γ receptor and incubated for four days. The cell supernatant was collected at the same time every day to quantify the DENV-2 RNA titer.

### Statistical Analysis

The survival rate differences among the different groups were analyzed by the Kaplan-Meier method. One-way analysis of variance (ANOVA) was used to compare differences among the groups. All data are shown as the mean ± SD. P values <0.05 were considered significant.

## Results

### Construction and Characterization of ZIKV zEDIII-rHF Nanoparticles

**Figure 1:**
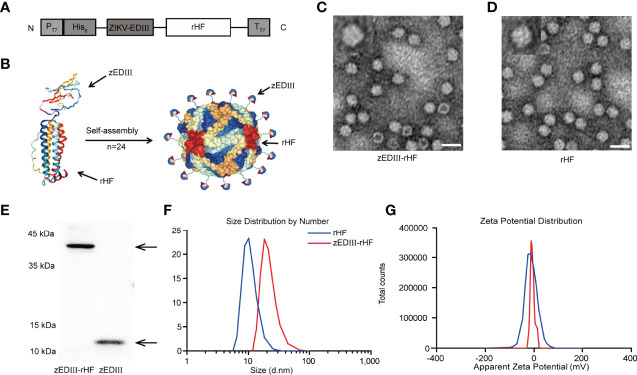
Construction and characterization of zEDIII-rHF nanoparticles. **(A)** Design of the zEDIII-rHF fusion protein: E protein domain III (zEDIII) was linked to the N-terminus of the human ferritin heavy chain (rHF). **(B)** 3D schematic diagram of zEDIII-rHF nanoparticles. **(C)** TEM image of zEDIII-rHF cages, bar=20 nm. **(D)** TEM image of rHF cages, bar=20 nm. **(E)** Western blot analysis of zEDIII and zEDIII-rHF. **(F)** Size distribution analysis of zEDIII-rHF nanoparticles *via* DLS showing an average diameter of 18.07 nm, which was larger than the diameter of rHF cages (mean = 12.92 nm). **(G)** Zeta potential distribution diagram for the cages. The average zeta potentials of zEDIII-rHF and rHF cages in assembly buffer were -15.27 ± 1.20 mV and -19.83 ± 1.07 mV, respectively.

To construct the nanovaccine, zEDIII was linked to the N-terminus of rHF (**Figure 1A**), and a nanoparticle displaying zEDIII was generated after rHF self-assembly. A diagram of the zEDIII-rHF nanoparticle is shown in **Figure 1B**. The zEDIII-rHF fusion protein was expressed in Escherichia coli and purified through Ni-chelating affinity chromatography and size-exclusion chromatography (SEC).Sodium dodecyl sulfate-polyacrylamide gel electrophoresis (SDS-PAGE) showed that the molecular weight of zEDIII-rHF was between 35-40 kDa, while those of rHF and zEDIII were 21 kDa and 14 kDa, respectively. zEDIII-rHF and zEDIII were further identified by Western blot analysis (**Figure 1E**). All the proteins were purified by removal of endotoxin to ensure that there were less than 0.001 endotoxin units per microgram of protein.

Transmission electron microscopy (TEM) showed that zEDIII-rHF could self-assemble into cage-like nanoparticles (**Figure 1C** and **Supplementary Figure 1**) in the same manner as rHF nanoparticles (**Figure 1D**). Dynamic light scattering (DLS) analysis was also performed to measure the diameter of self-assembled nanoparticles. The results indicated that the zEDIII-rHF cage had an average diameter of 18.07 nm, which was larger than the diameter of rHF cages (mean = 12.92 nm). This increase in nanoparticle diameter could be attributed to the presence of the exogenous protein, which indirectly reflected the modification of rHF cages (**Figure 1F**). We also measured the zeta potential of the zEDIII-rHF cage. As shown in **Figure 1G**, the zeta potential of zEDIII-rHF was -15.27 ± 1.20 mV), which was close to that of rHF (-19.83 ± 1.07 mV). This means that the display of zEDIII on the rHF cage did not affect nanoparticle stability.

### ZIKV zEDIII-rHF Activates Robust Humoral and Cellular Immunity in Mice

**Figure 2:**
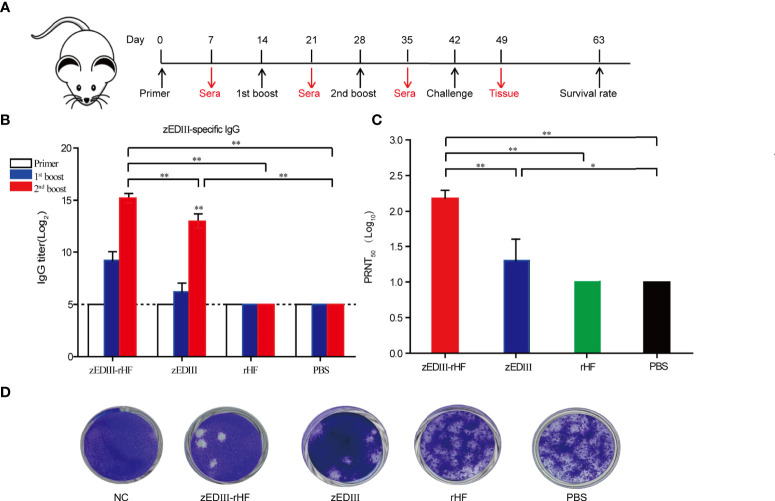
Humoral immunity induced by zEDIII-rHF in mice. **(A)** Scheme for immunization, virus challenge and sampling. **(B)** ELISA detection of zEDIII-specific IgG in sera after each immunization, n = 5. **(C)** Serum neutralizing antibody titers. Samples were diluted in a twofold gradient. **(D)** Number of viral plaques in Vero cell cultures (Vero cells were prepared in advance in a 24-well plate, and the ZIKV dose was 100 PFU). The data in 2b and 2c are shown as the mean and standard deviation (mean ± SD) analyzed by Student’s t-test; *P < 0.05, **P < 0.01. In **(B)**, the dotted line represents the limit of detection (LOD), which is the lowest dilution of the positive sample.

B6.129S2-Ifnar1^tm1Agt^ mice were used for immune analysis and subsequent virus challenge experiments, as this strain is a good animal model sensitive to ZIKV infection. Mice were intramuscularly (i.m.) vaccinated with zEDIII-rHF nanoparticles (10 μg), zEDIII (3.75 μg zEDIII molar equivalent), rHF nanoparticles (6.25 μg, rHF molar equivalent), or PBS. As shown in **Figure 2A**, the first vaccination time was considered day 0, and booster vaccinations were administered on days 14 and 28. Blood samples were collected to determine the zEDIII-specific IgG titer on days 7, 21 and 35. The results showed that the zEDIII-specific IgG titer was below the detection limit in all groups at the first vaccination. After boosting, the zEDIII-specific IgG titer increased dramatically in the zEDIII-rHF group, and this IgG titer was significantly higher than those of the other groups. zEDIII-rHF could induce a high zEDIII-specific IgG titer after the first booster immunization (>4000-fold), and zEDIII-specific IgG could be detected even when the serum samples were diluted more than 30000-fold after the second booster immunization, while the titers in the rHF and PBS groups were undetectable (**Figure 2B**). The final dilution in the zEDIII group was 8000-fold. In our study, the relationship between the immunological dose and the immune response was also investigated by vaccinating mice with different immunological doses of zEDIII-rHF. The results showed that an immunological dose of 10μg elicited a strong immune response in mice (**Supplementary Figure 3**).

After the final booster immunization, ZIKV-specific NAb titers were measured by a 50% plaque reduction neutralization test (PRNT_50_). Mice vaccinated with zEDIII-rHF were found to produce a high NAb titer (PRNT_50_ titer > 160), which was significantly higher than that of the zEDIII group (PRNT_50_ titer > 32). As controls, the mice in the rHF and PBS groups did not develop a detectable PRNT_50_ titer (PRNT_50_ titer<10-fold) (**Figures 2C, D**).

**Figure 3:**
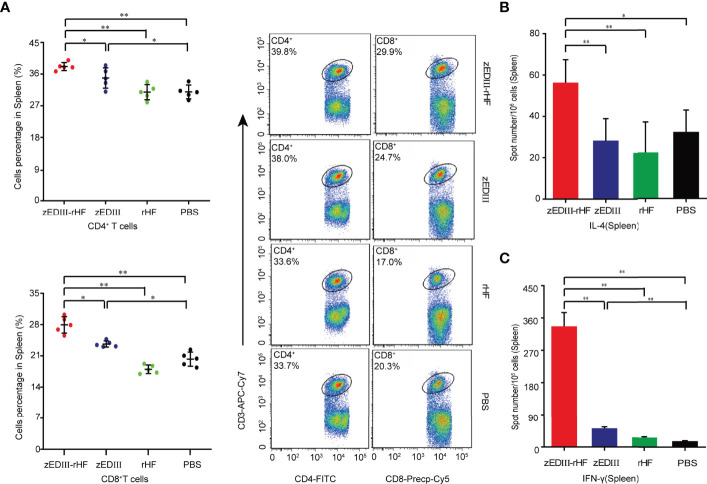
Cellular immunity induced by zEDIII-rHF in mice. Cellular immune responses were assessed on day 28 after the second booster immunization. **(A)** The percentages of CD4^+^ and CD8^+^ T cells in the spleen were determined by flow cytometry. **(B)** IL-4-secreting lymphocytes in the spleen, n = 3. **(C)** IFN-γ-secreting lymphocytes in the spleen were identified *via* an ELISPOT assay, n = 3. The data in 3a-3c are shown as the mean and standard deviation (mean ± SD) analyzed by Student’s t-test; *P < 0.05, **P < 0.01.

We evaluated zEDIII-rHF-activated cellular immunity, and splenic lymphocyte cells were analyzed by flow cytometry. Splenic lymphocytes were obtained using a lymphocyte isolation solution for flow cytometry. Notably, the CD4^+^ and CD8^+^ T cell percentages in the zEDIII-rHF group were significantly higher than those in the other groups (**Figure 3A**). As shown in **Figures 3B, C**, we further evaluated zEDIII-specific interleukin (IL)-4 and interferon-gamma (IFN-γ)-secreting lymphocyte numbers in the spleen with a cytokine enzyme-linked immunospot (ELISPOT) assay. The lymphocyte numbers in the zEDIII-rHF group (IL-4: 50 ± 6/10^6^ cells, IFN-γ: 335 ± 40/10^6^ cells) were significantly higher than those in the other groups. No significant differences were observed between the rHF or zEDIII group and the PBS group. In the zEDIII-rHF group, the IFN-γ-secreting lymphocyte numbers were much higher than the IL-4-secreting lymphocyte numbers.

### Protective Efficacy of zEDIII-rHF Nanoparticles Against ZIKV in Mice

**Figure 4:**
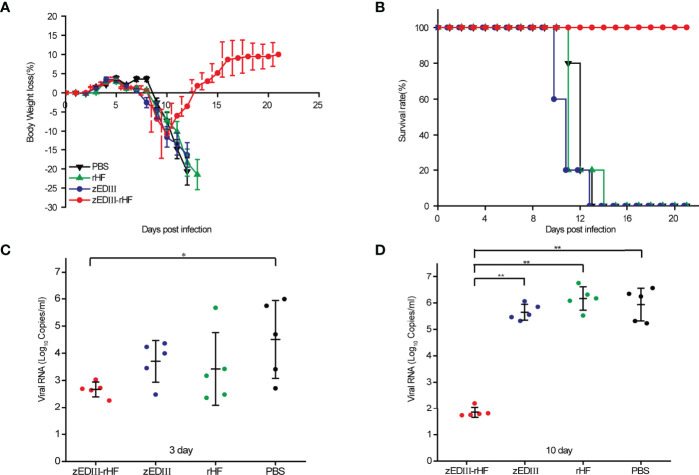
Protective efficacy against ZIKV infection. At two weeks postvaccination, mice were challenged with 20 μl of ZIKV (SZ-WIV01, 10× LD_50_) by foot pad injection. **(A)** Body weight changes of mice after challenge, n = 5. **(B)** Survival rates of mice after challenge, n = 5. **(C)** ZIKV RNA titers in the serum of mice 3 days after challenge, n = 5. **(D)** ZIKV RNA titers in the serum of mice 10 days after challenge, n = 5. The data in 4a-4d are shown as the mean and standard deviation (mean ± SD) analyzed by Student’s t-test; *P < 0.05, **P < 0.01.

Two weeks after final immunizations, B6.129S2-Ifnar1^tm1Agt^ mice were challenged with 20 μl of ZIKV (SZ-WIV01, 10× 50% lethal dose (LD_50_)) by foot pad injection. Body weight loss and survival data were collected for the next 21 days. Mice with body weight loss over 20% were assumed to be fatally infected and euthanized according to the study guidelines. Notably, in the zEDIII-rHF group, the weight of the mice recovered after a slight drop in body weight (**Figure 4A**), and all the mice in this group survived until the end point. (**Figure 4B**). Mice in the other groups, including the zEDIII group, died within 14 days postinfection, with body weight losses greater than 20%.

Viremia was examined on day 3 and day 10 after virus challenge by analyzing serum. **Figure 4C** shows that on day 3 postchallenge, a low titer of ZIKV RNA was detected in the zEDIII-rHF group, and this titer was significantly lower than those of the other groups. At day 10 postchallenge, almost no ZIKV RNA was detected in the zEDIII-rHF group, while high titers of ZIKV RNA (10^6.6^ copies/ml) could be detected in the control groups (**Figure 4D**).

**Figure 5:**
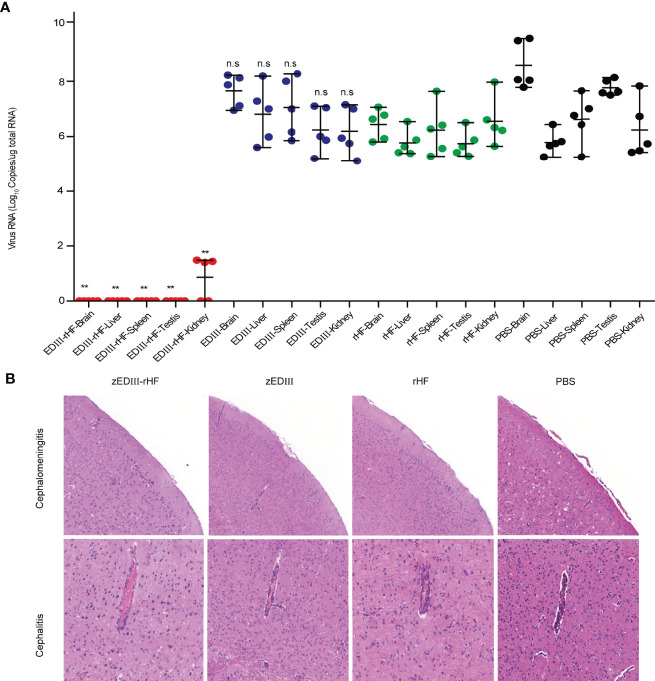
Mouse tissues were obtained for ZIKV RNA measurement and histopathological analysis on day 7 postchallenge. **(A)** ZIKV RNA titers in the brain, liver, spleen, testis, and kidneys. **(B)** Histopathological analysis of brain tissue sections. The data in 5a are shown as the mean and standard deviation (mean ± SD) analyzed by Student’s t-test (two-tailed; *P < 0.05, **P < 0.01 compared with the PBS-treated group), n = 5. ns, no significant difference.

The viral loads in different organs were also examined on day 7 after ZIKV challenge. Viral RNA was extracted from organs and measured by quantitative real-time PCR (qRT-PCR). As shown in **Figure 5A**, in the zEDIII-rHF group, the viral RNA titers were undetectable in the brain, spleen, liver, and testis, and only a very low titer could be detected in the kidneys. In contrast, high titers of viral RNA were detected in these organs in the other groups.

ZIKV infection causes cephalitis and cephalomeningitis. Thus, histopathological analysis of brain tissue sections was further carried out post-challenge. As shown in **Figure 5B**, no obvious disease symptoms could be detected in the brain of zEDIII-rHF-vaccinated mice. In contrast, mice in the zEDIII, rHF and PBS groups developed severe cephalitis and cephalomeningitis symptoms, congestion and inflammatory cuffs in small blood vessels were evident within the cerebral parenchyma, and cell shedding occurred in the cerebral cortex. This indicated that zEDIII-rHF vaccination could improve resistance to brain pathological symptoms caused by ZIKV infection.

### zEDIII-rHF Elicited an Immune Response Without ADE Activity for DENV-2 Infection

**Figure 6:**
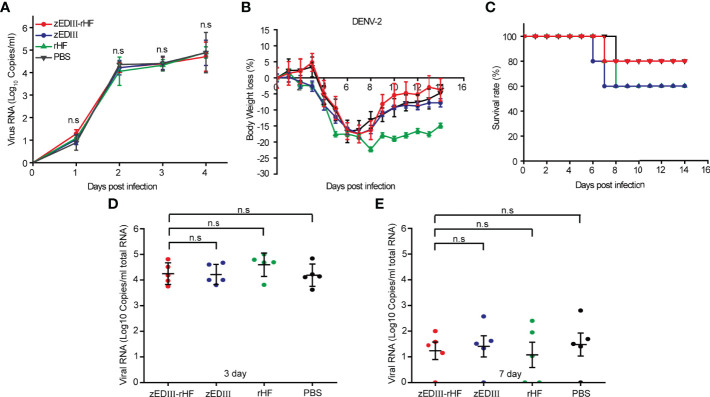
Analysis of ADE activity for DENV-2 infection after zEDIII-rHF vaccination. **(A)** Viral RNA titers in THP-1 cells incubated with DENV-2 TSV-01 mixed with mouse serum. **(B)** Body weight changes of mice after infection with DENV-2. **(C)** Survival rates of mice postinfection, n = 5. **(D)** DENV-2 RNA titers in mouse sera on day 3 postinfection, n = 5. **(E)** DENV-2 RNA titers in mouse sera on day 7 postinfection, n = 5.The data in 6a, 6d, 6e are shown as the mean and standard deviation (mean ± SD) analyzed by Student’s t-test; *P < 0.05, **P < 0.01. ns, no significant difference.

A key concern in the development of ZIKV vaccines is the risk of ADE of infections by heterologous flaviviruses (e.g., DENV). Therefore, we investigated whether the zEDIII-rHF nanoparticle-elicited immune response has ADE activity for DENV-2 infection. First, ADE of DENV-2 TSV-01 infection was examined in THP-1 cells with high expression of a specific Fc receptor. DENV-2 TSV-01 was mixed with diluted serum from vaccinated mice and inoculated into THP-1 cell cultures. Following incubation, viral RNA was detected by qRT-PCR. The result is shown in **Figure 6A**. The DENV-2 RNA level increased with time, but there were no significant differences between the zEDIII-rHF-vaccinated group and the other groups. This meant that the zEDIII-rHF nanoparticle-elicited antibodies did not enhance DENV-2 infection of THP-1 cells.

Additionally, after zEDIII-rHF vaccination, mice were challenged with 100 μl of DENV-2 (10^3^ 50% tissue culture infectious dose (TCID_50_)) by intraperitoneal injection (35). Body weight loss and survival data were collected for the next 14 days (**Figures 6B, C**). Mice exhibiting body weight loss over 20% were assumed to be fatally infected and euthanized. The results showed that the body weight of mice in the zEDIII-rHF group showed the same trend as that in the zEDIII, rHF and PBS control groups. There were no significant differences in body weight loss or the survival rate between the zEDIII-rHF group and the other control groups. Mouse sera were collected on day 3 and day 7 after DENV-2 challenge to examine viremia. The results showed that the viral titers of DENV-2 in the zEDIII-rHF group were comparable to those in the zEDIII, rHF and PBS control groups (**Figures 6D, E**). zEDIII-rHF vaccination did not promote DENV-2 replication in mice. Taken together, these results showed that zEDIII-rHF elicited an immune response that did not cause ADE of DENV-2 infection.

## Discussion

In view of the ZIKV epidemic and its clinical burden, there is an urgent need to develop an effective ZIKV vaccine. Herein, we developed a nanovaccine by displaying zEDIII on self-assembled rHF nanoparticle. The zEDIII-rHF nanovaccine combined the advantages of the ZIKV antigen zEDIII and the ferritin nanoplatform. Our results showed that zEDIII-rHF nanoparticles induced robust humoral and cellular immune responses, conferred complete protection, and did not induce ADE of DENV-2 infection.

zEDIII is the domain of the ZIKV E protein, which can induce host antibody responses with neutralizing activity (36). More importantly, zEDIII-specific antibodies do not show ADE activity, while ZIKV E protein domain I/II (zEDI/zEDII)-specific antibodies induces ADE activity which leads to enhancement of DENV infection (37). Thus, zEDIII is a promising vaccine candidate that is better than other forms, including E protein subunit vaccines (38, 39). However, the immunogenicity of zEDIII is relatively low, and zEDIII immunization could not control viremia or protect mice against ZIKV infection (40). In our experiment, all of the mice immunized with zEDIII at a dose comparable to the dose achieved by zEDIII-rHF nanoparticle administration died following ZIKV challenge (**Figure 4C**). Notably, zEDIII-rHF immunization completely protected mice against lethal ZIKV challenge. In zEDIII-rHF-immunized mice, there was also no cephalitis or other disease symptoms, and almost no viral RNA was detected at 7 days after ZIKV challenge. These results are amazing, making the approach of combining zEDIII with the rHF nanoplatform noteworthy.

The high protective efficiency of zEDIII-rHF nanoparticles should be attributed to their induction of robust immune responses. In our study, zEDIII-rHF evoked a high level of zEDIII-specific IgG, which has been demonstrated to play an important role in protective immunity (6, 41, 42). In the absence of any adjuvant, 10 μg of zEDIII-rHF elicited high zEDIII-specific IgG (log titer >4.5) and ZIKV-specific NAb titers (PRNT50 titer >160). In contrast, zEDIII elicited lower zEDIII-specific IgG and NAb titers. These results showed that displaying the zEDIII antigen on the ferritin nanoparticle significantly enhanced the immunogenicity of this antigen. More excitingly, zEDIII-rHF evoked a robust cellular immune response. In our experiment, zEDIII-rHF vaccination elicited high levels of CD4+ and CD8+ T cells. It has been reported that the ZIKV-specific CD4+ T cell response is critical for B cell activation and subsequent antibody maturation, playing a key role in resistance to primary ZIKV infection (43–45). CD8+ T cells protect the body against ZIKV infection mainly *via* cytolytic activity to kill infected cells and decrease virus-induced lymphocyte infiltration into the central nervous system (46–48). The strong CD8+ T cell response most likely contributed to eliminating virus-infected cells and preventing brain disease development. These findings should be of great significance in efforts to resist ZIKV infection and reduce symptoms.

In our study, zEDIII-rHF immunization induced significantly higher numbers of IFN-γ- and IL-4-secreting lymphocytes in the spleen of mice than did control immunizations. This indicated that T cell responses including Th1-type and Th2-type responses were efficiently induced by zEDIII-rHF nanoparticles. There were many more IFN-γ-secreting lymphocytes than IL-4-secreting lymphocytes, indicating that zEDIII-rHF nanoparticles induced Th1 type-biased responses (49). The Th1 response has been shown to be effective and preferable for controlling viral infection (50, 51), which provides a further explanation for the strong protection mediated by zEDIII-rHF vaccination. Notably, in our experiment, zEDIII without an adjuvant could not evoke T cell responses, although a certain level of zEDIII-specific IgGs could be induced, which could be one of the reasons why zEDIII did not protect against lethal ZIKV challenge.

zEDIII-rHF immunization did not show ADE activity for DENV-2 infection. ADE is an important concern for the development of vaccines against ZIKV and other flaviviruses. Abrogating or minimizing ADE has been pursued but is extremely challenging. ADE occurs because cross-reactive but subneutralizing antibodies facilitate viral binding and entry into Fc receptor-bearing cells and increase the virulence of infectivity (52). It has been reported that antibodies against EDI/II are cross-reactive and poorly neutralizing and can mediate heterologous ADE (53). Most current ZIKV vaccine candidates, such as live attenuated ZIKV and inactivated virus, and candidates based on ZIKV prM-E use the full-length ZIKV E protein, and thus, they have the risk of inducing ADE of heterologous flavivirus infections (54). Fortunately, antibodies against the zEDIII epitope are ZIKV-specific and neutralizing and thus do not induce ADE (16, 39). Indeed, in our study, zEDIII-rHF vaccination induced ZIKV-specific NAbs and did not exhibit ADE activity for DENV-2 infection. Therefore, the zEDIII-rHF nanoparticle provides a good vaccine candidate to abrogate ADE, which makes great progress in the safety of vaccines for ZIKV and other flaviviruses.

As a kind of recombinant nanoparticle, zEDIII-rHF can be easily produced in large quantities in *E. coli*. This provides another advantage for the zEDIII-rHF nanoparticle. The nanoparticle eliminates the need to produce viruses or virus-like particles (VLPs) in mammalian cells, and its robust production makes it competitive in industrial and commercial applications. In addition, during vaccination, no side effects, such as changes in body weight, mobility, or fur, were found in zEDIII‐rHF-immunized mice. No callosity or ulceration was observed at the injection site. The absence of any additional adjuvants during the immunization process also greatly reduces the risk of allergic reactions (55). At present, some ferritin carrier vaccine candidates have been shown to be safe and immunogenic in clinical trials (NCT03186781 & NCT03814720) (56). These results demonstrate that ferritin-based vaccines are safe and offer hopeful prospects for the use of the zEDIII‐rHF nanoparticle in biomedical applications. Thus, our new zEDIII-rHF nanovaccine shows superior properties including high immunity, complete protection, lack of ADE and robust production.

## Conclusions

We investigated a novel nanovaccine comprising ZIKV zEDIII assembled on ferritin nanoparticles. The zEDIII-rHF nanoparticle formulation induced strong humoral and cellular immune responses in the absence of any adjuvant and conferred complete protection against lethal ZIKV infection. Additionally, zEDIII-rHF immunization did not induce ADE of DENV-2 infection. The high immunity and protection of zEDIII-rHF, its lack of ADE and robust production indicate that zEDIII-rHF is a promising vaccine candidate against ZIKV.

## Data Availability Statement

The data that support the findings of this study are available from the corresponding authors upon reasonable request.

## Ethics Statement

The animal study was reviewed and approved by The Animal Care Committee of Wuhan Institute of Virology.

## Author Contributions

ZC conceived and designed the project; HR, MQ, JP, YS and BZ performed the experiments; HR, JG, XZ, and WL analyzed the data; and HR and ZC wrote the paper. All authors contributed to the article and approved the submitted version.

## Funding

This work was supported by the Strategic Priority Research Program of the Chinese Academy of Sciences (No. XDB29050201)、the National Natural Science Foundation of China (Nos. 31925025, 91743108, and 21727816) and the Key Research Project of Frontier Science of the Chinese Academy of Sciences.

## Conflict of Interest

The authors declare that the research was conducted in the absence of any commercial or financial relationships that could be construed as a potential conflict of interest.

## Publisher’s Note

All claims expressed in this article are solely those of the authors and do not necessarily represent those of their affiliated organizations, or those of the publisher, the editors and the reviewers. Any product that may be evaluated in this article, or claim that may be made by its manufacturer, is not guaranteed or endorsed by the publisher.
