# Supplementary material for: Self-Assembling Nanovaccine Confers Complete Protection Against Zika Virus Without Causing Antibody-Dependent Enhancement
Source: Front Immunol. 2022 May 9;13:905431. doi: 10.3389/fimmu.2022.905431 (PMC9124840; doi:10.3389/fimmu.2022.905431)
Supplement: Supplementary file 1 [file DataSheet_1.docx]

**Self-assembling Nanovaccine Confers Complete Protection Against Zika Virus Without Causing Antibody-Dependent Enhancement**

Heng Rong^1,2^, Mi Qi^1,2^, Jingdi Pan^1,2^, Yuhan Sun^1,2^, Jiawang Gao^1,2^, Xiaowei Zhang^1^, Wei Li^1^, Bo Zhang^1^, Xian-En Zhang ^3^, and Zongqiang Cui^1^*

Author affiliations

1 State Key Laboratory of Virology, Wuhan Institute of Virology, Center for Biosafety Mega-Science, Chinese Academy of Sciences, Wuhan 430071, People's Republic of China.

2 University of Chinese Academy of Sciences, Beijing 100049, People's Republic of China.

3 Faculty of Synthetic Biology, Shenzhen Institutes of Advanced Technology, Chinese Academy of Sciences, Shenzhen 518055, China.

* Corresponding author: E-mail: [czq@wh.iov.cn](mailto:czq@wh.iov.cn).

**Supplement**

**
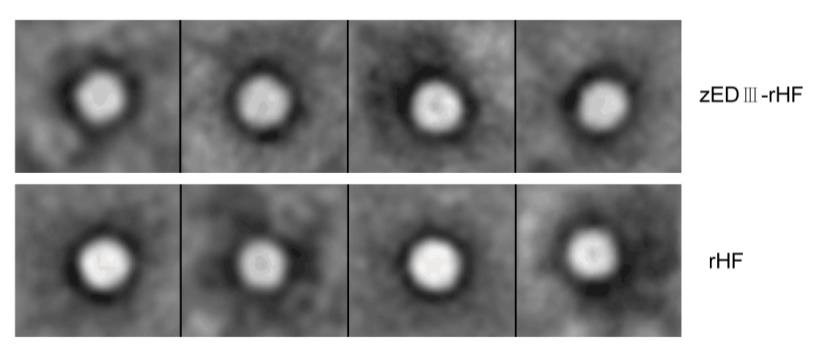
**

**Supplementary Figure 1. 2D classification of TEM images of zEDIII-rHF and rHF nanoparticle.**

The TEM images of negatively stained particles were analyzed with RELION software, and the classified particles show four directions of observation. The zEDIII-rHF associated 2D class averages were obtained from 176 particles. The rHF associated 2D class averages were obtained from 100 particles.


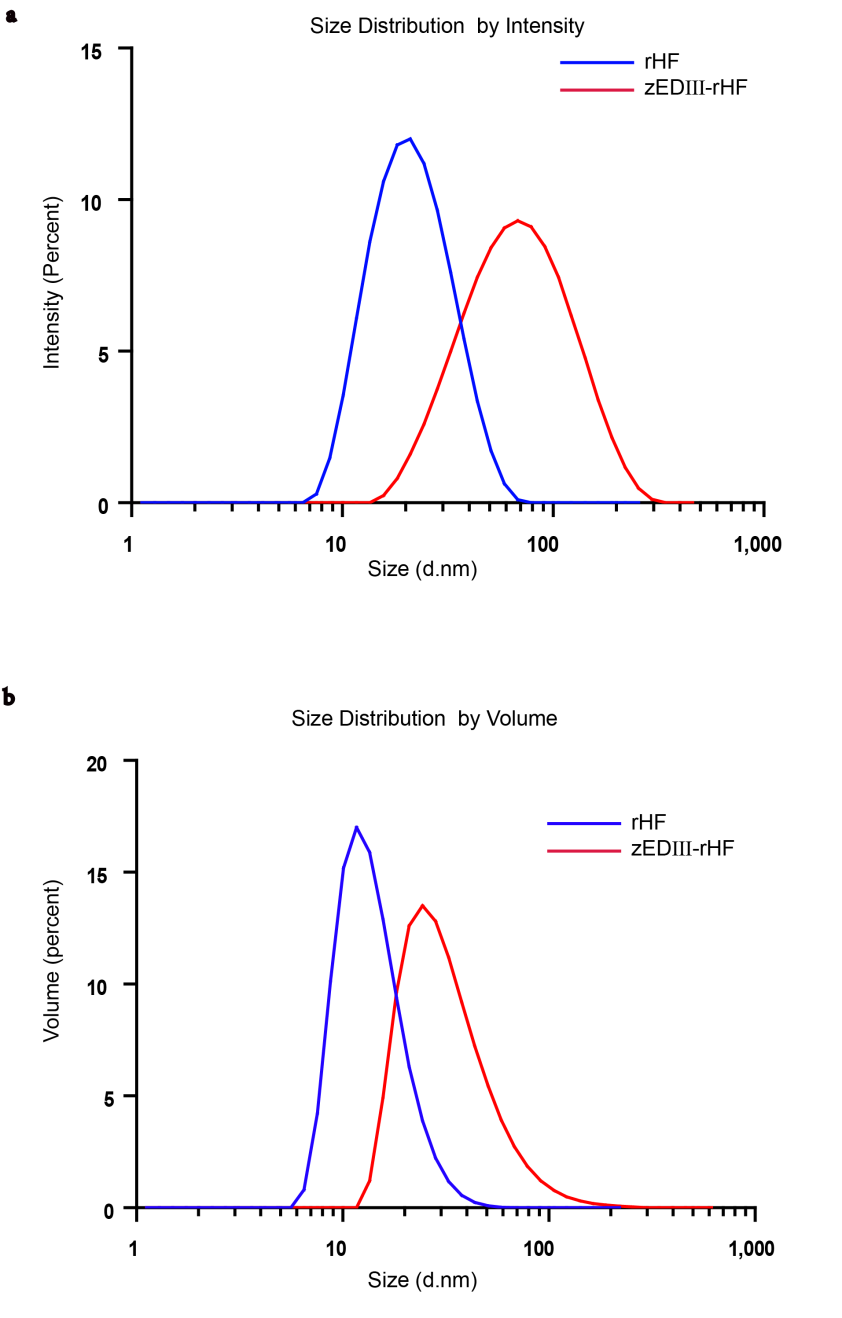


**Supplementary Figure 2. zEDIII-rHF and rHF nanoparticle characterization.** a) DLS analysis of the intensities of zEDIII-rHF and rHF nanoparticles. b) DLS analysis of the volumes of zEDIII-rHF and rHF nanoparticles.


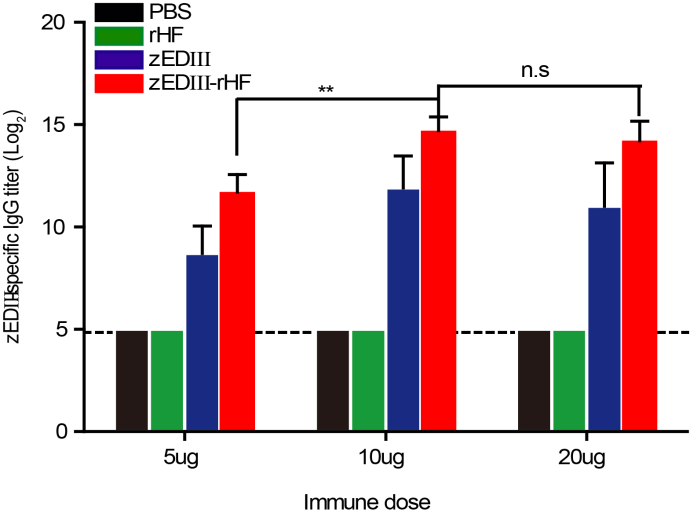


**Supplementary Figure** 3. **Dose-dependent experiment.** Mice were vaccinated with different immunological doses. Serum samples were collected 7 days after the final booster immunization. The data in the figure are shown as the mean and standard deviation (mean ± SD) analyzed by Student’s t-test; **P < 0.05, **P < 0.01* among the three groups).
